# Mister Mary Somerville: Husband and Secretary

**DOI:** 10.1007/s00283-020-09998-6

**Published:** 2020-08-03

**Authors:** Brigitte Stenhouse

**Affiliations:** School of Mathematics and Statistics, Faculty of STEM, The Open University, Walton Hall, Milton Keynes, MK7 6AA UK

Mary Somerville’s life as a mathematician and savant in nineteenth-century Great Britain was heavily influenced by her gender; as a woman, her access to the ideas and resources developed and circulated in universities and scientific societies was highly restricted. However, her engagement with learned institutions was by no means nonexistent, and although she was 90 before being elected a full member of any society (Società Geografica Italiana, 1870), Somerville (Figure 1) nevertheless benefited from the resources and social networks cultivated by such institutions from as early as 1812. A key intermediary between Somerville and these societies was her husband, Dr. William Somerville, whose mediation was vital to her access to knowledge and her subsequent career as a scientific author. In this paper we will consider how spousal cooperation enabled the overcoming of gendered barriers to scientific institutions in the nineteenth century.

**Figure 1:**
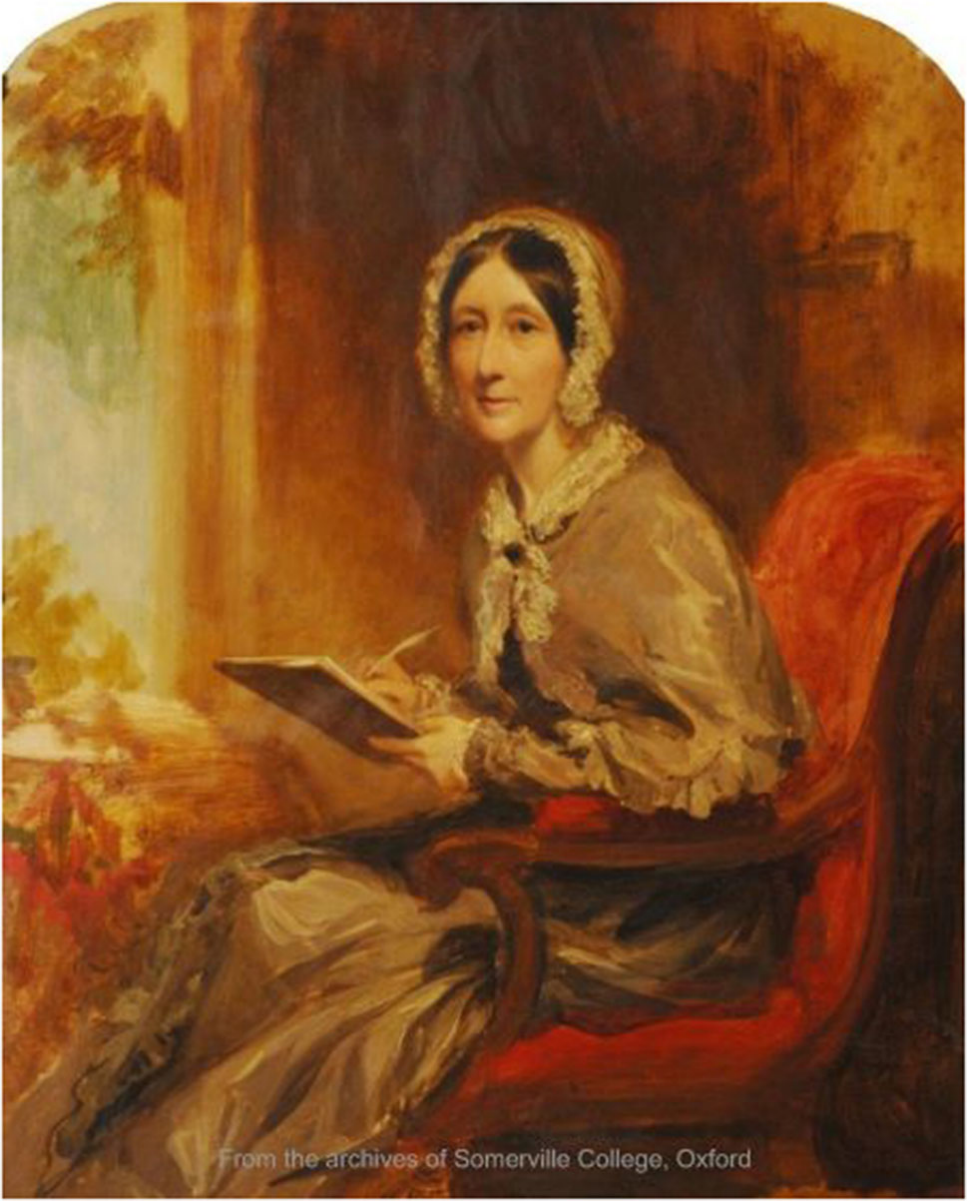
Self-portait of Mary Somerville. Courtesy of Somerville College, University of Oxford.

In considering the role of women in science and mathematics, we see that scientific societies and institutions usually play an exclusionary role. Women in Britain had no access to higher education until the founding of Bedford College, London, in 1848, and to this day, there has been no female Astronomer Royal (a prestigious post for a nineteenth-century mathematician). Although no scientific learned society had a formal statute barring women during Somerville’s lifetime, there was nonetheless a great reluctance even to allow women into the buildings, never mind to endow them with the rights of members. Except for the visit of the prolific author Margaret Cavendish in 1667, the Royal Society of London did not invite women into their hallowed halls until 1876, with the commencement of their second *conversazione* [15, 163], which women were permitted to attend.^1^ As late as 1886, on the nomination of Isis Pogson as a fellow, the Council of the Royal Astronomical Society chose to interpret their constitution as explicitly excluding women [12].^2^ National societies that aimed to promote mathematics specifically were not founded until near the end of Somerville’s life, namely the London Mathematical Society in 1865 and the Société Mathématique de France in 1872, and again there was a significant delay before women were elected members.^3^

However, focusing too heavily on membership alone can distort our understanding of the influence that these institutions exerted. It can furthermore lead to underestimating the role played by informal knowledge exchange through letter correspondence and polite sociability,^4^ activities that took place adjacent to the institutions themselves.^5^ As Charles Babbage (1791–1871) noted in his 1830 polemic against the Royal Society, only 109 out of 714 fellows had contributed a paper to the *Philosophical Transactions of the Royal Society* (*Phil. Trans.*) [4, 154–155], while Caroline Herschel (1750–1848), who was never affiliated even as an honorary member, had thrice published descriptions of her discoveries of new comets.^6^ For women, membership itself could be the least significant interaction with these institutions.

## Mary Somerville as an Honorary Member

Mary Somerville (1780–1872, née Fairfax^7^) was a Scottish mathematician and scientist who was remembered on her death as “one of the most distinguished astronomers and philosophers of the day” [31] in [46, Vol. 1]. In her lifetime she published four books, which cumulatively went through 17 editions (not including the many pirated editions published in the United States of America), as well as appearing in translation in French, German, and Italian. Somerville also had papers published in the *Philosophical Transactions* and the *Quarterly Review*, and extracts from her letters were published in the *Comptes Rendus de l’Académie des Sciences* and the *Edinburgh New Philosophical Journal* [46].

Although her gender precluded her from attending university or holding full memberships in scientific academies relevant to her mathematical and scientific research, Somerville was awarded multiple honorary memberships. The earliest of these were in recognition of her first book, *Mechanism of the Heavens*, published in 1831 [43]. A translation and adaptation of Pierre-Simon Laplace’s formative *Traité de mécanique céleste* [23], this book in four parts played a key role in the circulation of calculus in Great Britain and was recommended to students studying at Cambridge University in the 1830s [9], [47, p. 172].

**Figure 2:**
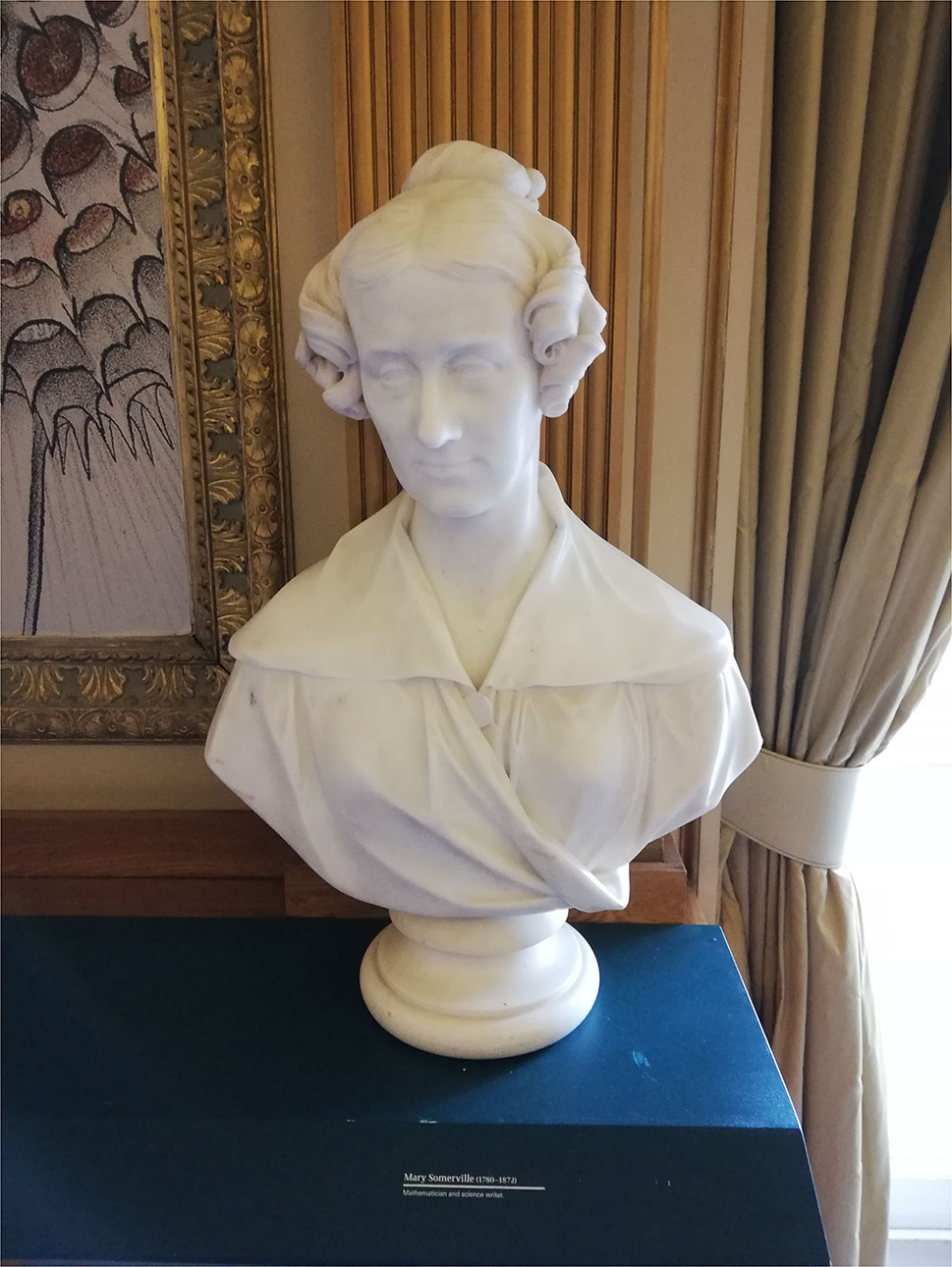
Marble bust of Mary Somerville by Francis Chantrey, Royal Society of London. Photograph by the author, reproduced by permission of the Royal Society. Photograph by the author, reproduced by permission of the Royal Society.

The Naval and Military Library and Museum of London was the first society to list Somerville as an honorary member, on 21 September 1832. This was followed in 1834 by election to the Société de Physique et d’Histoire Naturelle de Genève and the Royal Irish Academy, Dublin. Mary Somerville and Caroline Herschel were the first women to be elected honorary members of the Royal Astronomical Society (RAS), in February 1835, and later that year, Somerville could add a certificate of honorary membership of the Bristol Philosophical and Literary Society to her collection [47, 172–176].^8^ Although Mary Somerville was never elected a Fellow of the Royal Society, in 1832, sixty-four fellows pledged £156.10 to commission a marble bust of her to be placed in the society’s meeting room (see Figure 2), in order to pay tribute to “the powers of the female mind, and at the same time establish an imperishable record of the perfect compatibility of the most exemplary discharge of the softer duties of domestic life, with the highest researches in mathematical philosophy.”^9^

These honorary memberships appear not to have benefited Somerville in any meaningful way. Payment of an admission fee and subsequent yearly subscription gave members of the RAS access to the society’s meeting rooms and the right to append the letters FRAS after their name [4, 43]. In the letter from Augustus De Morgan (1806–1871, professor of mathematics at University College London and secretary of the RAS) in which he informs Somerville of her election to honorary membership, there is no suggestion that she is liable for this admission cost. Nor is her entitlement to these privileges made clear.^10^ Indeed, although she was certainly aware of her honorary election to the RAS when it occurred, and later mentioned the election in her autobiographic *Personal Recollections* [47, 173], when visiting the society in 1844 she claimed to be unaware that the election had even taken place!^11^ Whether this was because she had genuinely forgotten or because she felt unable to assert her right to enter the building on the basis of her own membership is impossible to say; nevertheless, this clearly suggests that she had not made free use of the space since her election in 1835. None of the other societies that bestowed honorary membership on Somerville were based in London (where she resided until 1838), so even had she wanted to attend meetings or make use of the facilities, that would have been expensive and difficult. Similarly, Somerville did not advertise her affiliations with learned societies by appending the appropriate letters to her name when signing her correspondence, nor in the title pages of her publications, where she appeared merely as “Mrs Somerville” until 1835 and “Mary Somerville” from then on.^12^

As we will see, thanks in large part to her husband, long before her honorary memberships Somerville had already been successfully circumventing the barriers she faced to engage with the communities centered on the learned academies in London, Paris, and Geneva.

## Society Memberships of Dr. William Somerville

Mary Somerville married her cousin Dr. William Somerville (1771–1860) in May 1812.

**Figure 3:**
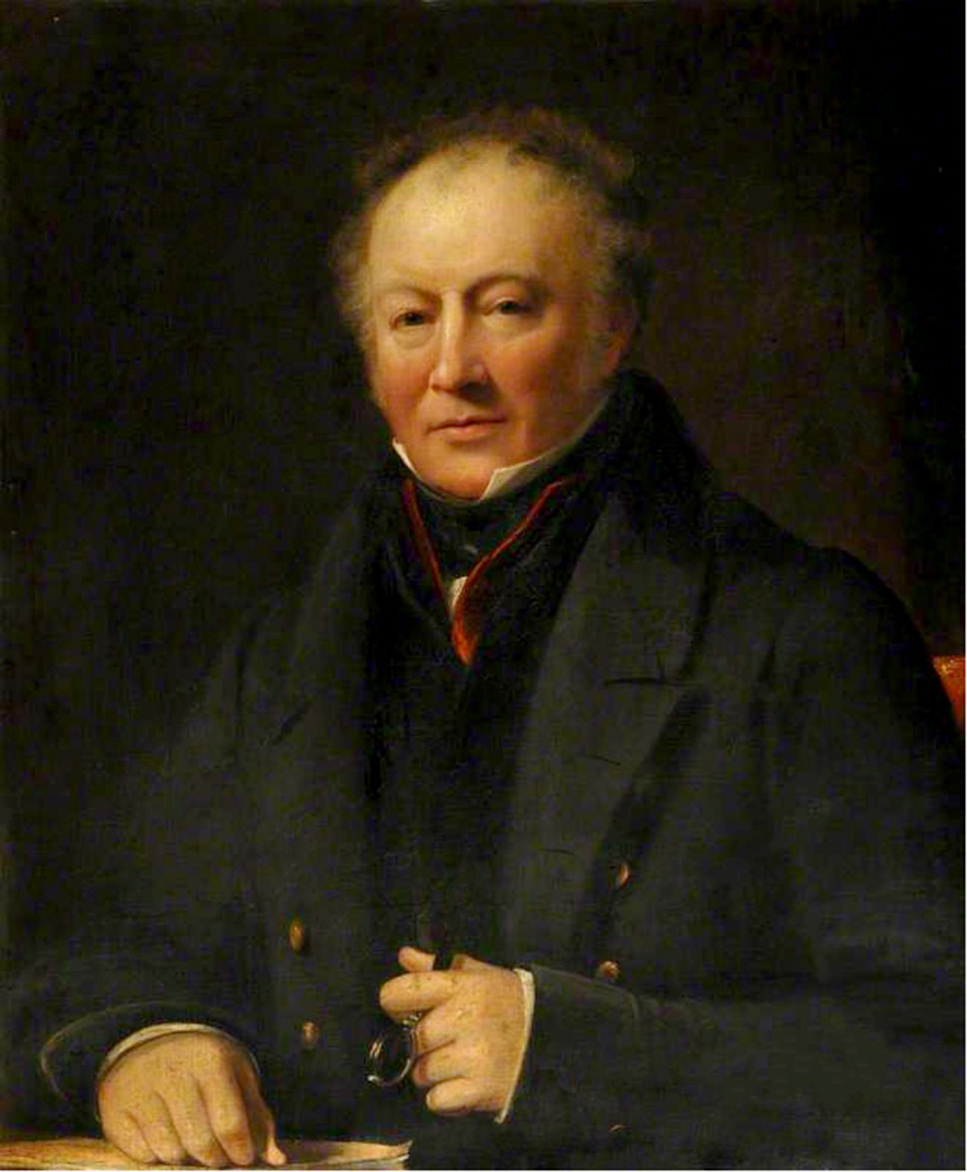
Portrait of William Somerville. Oil on Canvas. Courtesy of Somerville College, University of Oxford.

Throughout his life, William was interested in natural philosophy, although, as was still usual at the time he treated it, more as a “gentlemanly pursuit” than a serious vocation. As an army surgeon, William was posted to South Africa in the 1790s, where he wrote of his interactions with the local population, as well as descriptions of the local wildlife (Fig. 3).^13^ He was later posted to Malta and Canada before returning to Scotland in 1811, when he proposed to Somerville. After a brief time in Portsmouth, the newly married couple settled in Edinburgh in 1813, when William was appointed head of the Army Medical Department in North Britain [34, 6–8].

The social connections that the Somervilles made while in Edinburgh were vital to their later entry into polite scientific society in London and during their tours of Europe in 1817, 1824, and 1831. In January 1813, William was elected an Ordinary Member^14^ of the Royal Society of Edinburgh (RSE), having been proposed by John Playfair (1748–1819), who was then the holder of the chair in natural philosophy at the University of Edinburgh and secretary of the RSE [2, 542], [50, 869].^15^ During the same election, zoologist Georges Cuvier (1769–1832) and mathematician Pierre-Simon Laplace (1749–1827) were elected as honorary members, both of whom the Somervilles would later meet in Paris. In 1816, just before moving to London on William’s appointment as a principal inspector of the Army Medical Board, the Somervilles became acquainted with Leonard Horner (1785–1864, a factory inspector and FRSE from 1816), possibly through their RSE connection. Horner played a key role in the Somervilles’ new life in London; through a letter of introduction, he facilitated their acquaintance with Alexander (1770–1822) and Jane Marcet (1769–1858), a physician and scientific author respectively. In his letter, Horner described William Somerville as “a very good fellow, & his wife a very interesting woman. She is a person of extraordinary acquirements, particularly in mathematics” [34, 12].

The Somervilles appear to have been welcomed into London scientific society with open arms [34, 12–14]. By December 1817, William Somerville had been elected a Fellow of the Royal Society, and Alexander Marcet was one of seventeen signatories on his certificate of election alongside mathematician John Herschel (1792–1871), Astronomer Royal John Pond (1767–1836), as well as chemists and future presidents of the society Sir Humphry Davy (1778–1829) and William Hyde Wollaston (1766–1828).^16^ The certificate notes William’s acquirements in natural history and mineralogy, and that he was by this point already a fellow of the Linnean Society and the Geological Society.^17^

We highlight here that it was only William’s acquirements that made him eligible for membership in the Royal Society; but what of Somerville’s acquirements? Playfair, who nominated William for membership of the RSE, was certainly aware of her mathematical aptitude, as together they had discussed Laplace’s *Mécanique céleste*, and in June 1812, he wrote a letter of introduction for the Somervilles, addressed to William Herschel, in which he claimed that Somerville was “distinguished by knowledge of the Mathematical Sciences rarely to be met with in men,” noting especially her studies in geometry, algebra, and astronomy [47, 81].^18^ A year earlier, Somerville had written to Playfair’s former mentee William Wallace (1768–1843, professor of mathematics at the Royal Military College) with a solution to a mathematical puzzle circulated in the *New Series of the Mathematical Repository*, which was subsequently published in the periodical and for which Somerville was awarded a silver medal; this led to a fruitful correspondence in which Wallace supported Somerville’s mathematical studies by setting questions and critiquing her solutions [48]. Somerville’s reputation for excellence became so widely known that in 1822, novelist Maria Edgeworth (1768–1849) described her as “the lady whom La Place mentions as the only woman in England who understands his works,”^19^ and in 1826, when Henry Brougham (1778–1868, first Baron Brougham and Vaux and founder of the Society for the Diffusion of Useful Knowledge) desired to commission a translation of *Mécanique céleste* into English, he claimed that if Somerville was unable to complete the work, then it would have to be left undone, as “none else can” [47, 161–162]. Furthermore, alongside knowledge of natural philosophy more broadly or employment in universities, being “conversant” in mathematics was used as justification for the election of 25 new fellows of the Royal Society during this time of Somerville’s increasing renown, and in 1823, Lewis Evans was elected purely for being “a Gentleman well skilled in Mathematics and Astronomy.”^20^ Therefore, the absence of Somerville’s nomination, to the Royal Society at least, was clearly an issue of gender.

Nevertheless, Somerville was by no means isolated from scientific societies, for she was able to engage in the polite sociability surrounding and connecting these closed institutions, which was a key component of scientific and mathematical activity. Moreover, William actively shared the benefits of his memberships, and, depending on the situation, took on the roles of Somerville’s chaperone, secretary, representative, or even literary agent. We will investigate each of these in turn, to illuminate the ways in which Somerville’s engagement in mathematical and scientific communities was affected and improved through her husband’s assistance.

## William Somerville as Chaperone

On her marriage to William, Somerville’s social and geographical mobility was transformed, since with a husband who shared her scientific interests and enjoyment of polite company, she now had a constant companion and eager chaperone.

Although British women from the middle and upper classes had been global travelers since at least the early eighteenth century, it was very rare for a woman to travel alone. Very often, a woman would travel with her spouse as a companion or as a collaborator taking an active part in observation and collecting, depending on the purpose of the travel; without a family member to act as chaperone, women were otherwise dependent on finding paid servants or local guides willing to accompany them [29, 29].^21^ Travel costs were prohibitive enough to the Somervilles even without the added cost of paying for a maid to act as a companion and provide childcare on the go, and in 1832, Somerville lamented that she was forced to be “stationary all summer [because] moving is so expensive” [34, 94].^22^

The importance of a chaperone is underlined in Somerville’s letters from Francis Jeffrey (1773–1850, editor of the *Edinburgh Review*), in which he implored her to attend the 1834 annual meeting of the British Association for the Advancement of Science (BAAS), which took place in her former home city, Edinburgh. He expressed his great disappointment that she was not intending to travel north for the meeting, both for the personal loss of her good company and for the fact that the first Scottish meeting of the BAAS would be deprived of the honor of her attendance. Jeffrey acknowledged the inconvenience to William to be so far from London at that time as the reason for Somerville’s intended absence, and asked,

if the inconvenience is insurmountable, should not *you* come without him? If I were in your neighbourhood I should whisper this in your private ear, in the most seductive terms ... the Dr did allow you to stay Heaven knows how many months in the profligate Paris without him. I cannot but hope that he may consent your being as many weeks in our moral Edinburgh.^23^

That Jeffrey should feel the need to convince Somerville to travel without her spouse in a “private seductive whisper” strongly suggests that he was aware that it would be a decision that could not be made lightly. Moreover, his recourse to the moral standing of Edinburgh makes clear that the difficulties and dangers lay not just in the travel itself (the journey from London to Edinburgh would have taken around 10 days by coach), but also in attending society and BAAS gatherings while in the city.^24^

With the accompaniment of her husband, Somerville was able to expand her circle of acquaintances beyond Edinburgh by traveling not only within the UK, but to France, Prussia, Switzerland, the Netherlands, and the Italian peninsula. Within a year of their wedding in 1812, the Somervilles traveled to Marlow (near London) to visit Somerville’s mentor William Wallace, with whom she had previously interacted only via letter. It was perhaps at this time that Wallace gave Somerville his copy of Joseph Louis Lagrange’s *Théorie des fonctions analytiques* and offered advice on which texts she should purchase for her personal mathematical library [47, 79].^25^ Wallace also escorted the newlyweds to Slough, where they met the astronomer William Herschel (1738–1822) and his son John Herschel, who was later a signatory on William Somerville’s certificate to election of the Royal Society and instrumental in the preparation of *Mechanism of the Heavens*.^26^

In 1817, the Somervilles embarked on a journey through France, Switzerland, and the Papal States. Letters of introduction to people of note who resided in travelers’ intended destinations were vital in facilitating entry into the local polite society [29, 48]. Having already met Jean-Baptiste Biot (1774–1862) and François Arago (1786–1853) in London, on arriving in Paris, the Somervilles gained easy access to the most prestigious learned institutions and became acquainted with many of the best-known philosophers of the day. During her two weeks in the city, Somerville heard papers read at the Institut de France, visited astronomer Claude Louis Mathieu (1783–1875) at the Paris Observatory, and received “the greatest attention” from Gabrielle Biot (1781–1851, a scientific translator and wife of Jean-Baptiste), who organized a dinner in order to introduce Somerville to “les personnes distinguees [sic],” including mathematician Siméon-Denis Poisson (1781–1840) and geographer Alexander von Humboldt (1769–1859).^27^

Near the end of their visit, the couple were hosted by Pierre-Simon Laplace at Arcueil; that Somerville was able to meet and impress the mathematician whose work she was so well known for having studied when few others in Britain were capable of doing so was invaluable to both her intellectual pursuits and her reputation. The claim that mathematicians benefit from discussing concepts and ideas with “colleagues” will be, I hope, uncontroversial, and although Somerville had previously benefited from such intellectual exchange through her aforementioned discussions of Laplace’s *Mécanique céleste* with John Playfair, in his 1808 review of the work, Playfair himself admitted to his own limited understanding of the advanced mathematics it contained [36, 275]. At dinner in Arcueil, Somerville engaged Laplace in discussions of his scientific works that were clearly not vacuous, since seven years later, he wrote to Somerville claiming that “the interest which you deign to take in my work flatters me all the more as there are few other readers and judges so enlightened” [18, 1250–1251].^28^ Moreover, he enclosed a copy of the fifth edition of his *Système du monde* for Somerville to add to her personal collection of mathematical texts, giving her the freedom to consult it at her leisure.^29^ This endorsement from Laplace compounded Somerville’s reputation as an expert mathematician and was echoed throughout contemporary accounts of her life, not only in the description given by Edgeworth above, but even showing up in the diary of Queen Victoria in 1838.^30^

Beyond an increased geographical mobility, Somerville’s marriage to William also increased her mobility within polite scientific society itself. On moving to London in 1816, the Somervilles took up residence at Hanover Square, in London’s fashionable West End, where they were well positioned to engage in the social calls and occasions that made up London society. In her *Personal Recollections*, Somerville recounts numerous instances of engaging in informal experiments or making observations in the homes and gardens of her friends. One such anecdote entails testing the power of a telescope by making observations of double stars (a pair of stars that appear close together and often require a powerful telescope to distinguish them individually) with Henry (1777–1835) and Mary Frances Kater (1784–1833) until the early hours of the morning. On their way home, the Somervilles noticed a light in the window of Thomas Young (1773–1829, author of an anonymous partial translation of *Mécanique céleste* [51]), and on ringing his bell, they were invited inside to see an Egyptian papyrus that Young had just identified as a horoscope. The dates and details of such stories as given by Somerville are often unreliable, but the impression remains (and is borne out in extant correspondence) that she was able to enjoy close personal connections as well as intellectual exchanges through her lively social life.

While there is little extant evidence of how Somerville was able to cultivate social connections with such a vast array of notable scientists and luminaries, it is likely that the Somervilles’ participation in scientific societies and institutions played a key role. Hanover Square was within walking distance of the Royal Institution (RI) on Albemarle Street, which soon after its founding in 1799 had been absorbed into the London social season with “subscribers” attending lectures in the same way that they would attend the opera or theater [47, 107], [25, 113]. Women were eligible for all levels of membership of the RI,^31^ and indeed, between 1800 and 1812, women often outnumbered men in the audiences of lectures, which covered scientific topics such as mechanics, chemistry, and botany, as well as painting, architecture, and poetry [25, 123–124]. While we know a little bit about William’s engagement with the RI, namely that he was listed as an annual subscriber in 1816 and later named on the “List of Managers of the Royal Institution” [34, 11,91], less is known about Somerville’s. A “Mrs Greig of Great Russell Street” subscribed to the RI in 1805, when Somerville lived in London with her first husband, and a “Mrs Somerville of Hanover Square” subscribed to the lectures in 1825.^32^ Somerville does not allude to her RI membership in 1805, but does recall attending the lectures, frequently with William, on her return from traveling in Europe in 1818 [47, 107].

Founders of scientific societies consciously recognized the importance of facilitating social connections; when the Royal Society of Edinburgh was founded in 1783, one of its three objectives was to provide a “personal and informal” social space for fellows (the other two being to assemble a library and publish a periodical) [8, 8]. The Geological Society (of which William was a member) noted the importance of connecting those with scientific interests as “the remarks which are made by separate inquirers, however interesting in themselves, are less valuable from being unconnected” [38, v–vi]. Thus as a member of multiple societies, as well as gentlemen’s clubs such as the Athenaeum and exclusive dining clubs such as the Pow-Wow Club, William was well placed to meet the brightest stars in British science [34, 32].

## William Somerville as Representative

**Figure 4:**
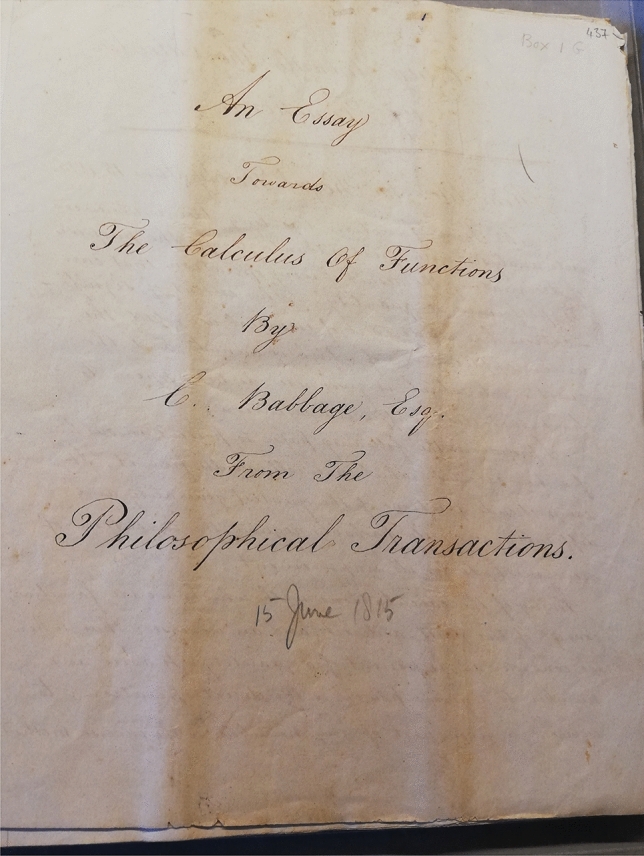
Front matter of manuscript copy of a *Phil. Trans.* paper by Charles Babbage.

Nonetheless, even with such an able and willing chaperone, there were doors that remained closed to Somerville. In an undated letter from mathematician Charles Babbage, Babbage gives the details of a dinner to which both William and Somerville are expected, and then the time and location of the inaugural meeting of the Statistical Society, which was cofounded by Babbage in 1834.^33^ Babbage and Somerville were well acquainted by 1834 and had shared a distinctly mathematical discourse; within their extant correspondence we see Somerville invited to Babbage’s house to view his “calculating machine,” and Babbage offering advice during the preparation of *Mechanism of the Heavens*. Multiple letters mention the sharing of mathematical papers such as John Herschel’s and Augustus De Morgan’s articles in the *Encyclopædia Metropolitana*, and manuscripts of five of Babbage’s own articles can be found in the Mary Somerville Papers (see Figure 4).^34^ Meanwhile, the correspondence between Babbage and William focuses for the most part on social engagements. Yet it was only to William that the invitation to the inaugural meeting of the Statistical Society was extended.

Therefore, within the physical spaces of the scientific societies, William was required to act as Somerville’s representative and advocate. One of the most visible and significant instances of this took place in February 1826, when William communicated Somerville’s paper “On the magnetizing power of the more refrangible solar rays” to the Royal Society. When subsequently printed in the *Phil. Trans.*, this paper was Somerville’s first publication under her own name [42].

According to their daughter, who edited Somerville’s *Personal Recollections*, William would visit libraries of the learned societies on Somerville’s behalf to source books she required [47, 85]. This is corroborated in the lending records of the Royal Society, in which his name appears 15 times between 1825 and 1840: in 1828, he took out two volumes of Roger Long’s *Astronomy, in five books* [26]; in 1832, he borrowed Poisson’s *Nouvelle théorie de l’action capillaire*, Biot’s *Précis élémentaire de physique expérimentale*, and volume 106 of the *Phil. Trans.*, which contained mathematical papers by both Babbage and John Herschel from their time in the Analytical Society;^35^ entries in 1834 include Volume 9 of the *Philosophical Magazine* and Volume 3 of the *Mémoires d’Arcueil*; and finally, in 1837, William borrowed Volumes 1 to 13 of the *Comptes Rendus*. Therefore, Somerville had access to expensive texts, many of which were published overseas and would otherwise have been very difficult to source. Regardless of whether William did in fact borrow these books specifically for Somerville, they would almost certainly have been available for her to read at home. Moreover, during 1832, 1834, and 1837, Somerville was in the process of preparing successive editions of her second book, *On the Connexion of the Physical Sciences* (see below), and the texts borrowed by William would have been indispensable in preparing and revising that work.

Communication of ideas at this time did not rely solely on printed texts; information was passed to Somerville within epistolary correspondence itself. The astronomer Francis Baily (1774–1884, cofounder and at that time a vice president of the RAS) wrote to William in February 1833 that he “should be most happy to answer Mrs. Somerville’s enquiries, relative to the compression of the Earth.”^36^ Although he felt he could not add anything to what Somerville already knew, Baily used the measurements of the Earth’s semiaxis and equatorial radius from George Biddell Airy’s 1830 paper on the “Figure of the Earth” to give an estimate of the compression of the Earth [1] and expressed his disappointment that those measurements did not make a closer match with the compression calculated from pendulum experiments. Baily concluded his letter by asking William to reassure Somerville that he would be “at all times most happy to communicate [to Somerville] any information in [his] power.”

Moreover, while at a society council meeting in March 1832, William Somerville, on behalf of his wife, solicited William Broderip (1789–1859, a magistrate, enthusiastic shell collector, and an original fellow of the Zoological Society) for information regarding plants of the Himalayas.^37^ The next day, Broderip wrote to Somerville directly to supplement the “few hints [he] was able to give [William] during council.”^38^ Broderip directs Somerville to John Gould’s *A Century of Birds from the Himalaya Mountains* [16]; lists twenty varieties of flora to demonstrate that the same genera (although different species) of flowers are found in both the Himalayas and the Alps; and begs Somerville to visit the nursery of a “Mr. Knight” before the end of spring in order to see his specimen of the Nepalese flower *Rhododendron arboreum* in bloom.

Therefore, although Somerville was not directly involved in the frequent comings and goings of the social clubs and learned societies of nineteenth-century London, through the active participation of her husband she was nonetheless able to engage with and benefit from the easy and informal exchange of information that took place there. Furthermore, similarly to her contemporaries, Somerville pursued mathematics alongside at least mineralogy, botany, and chemistry; this breadth of interests allowed for more meaningful engagement in a scientific community that placed little value on specialization or esoteric knowledge.^39^

## William Somerville as Secretary

**Figure 5:**
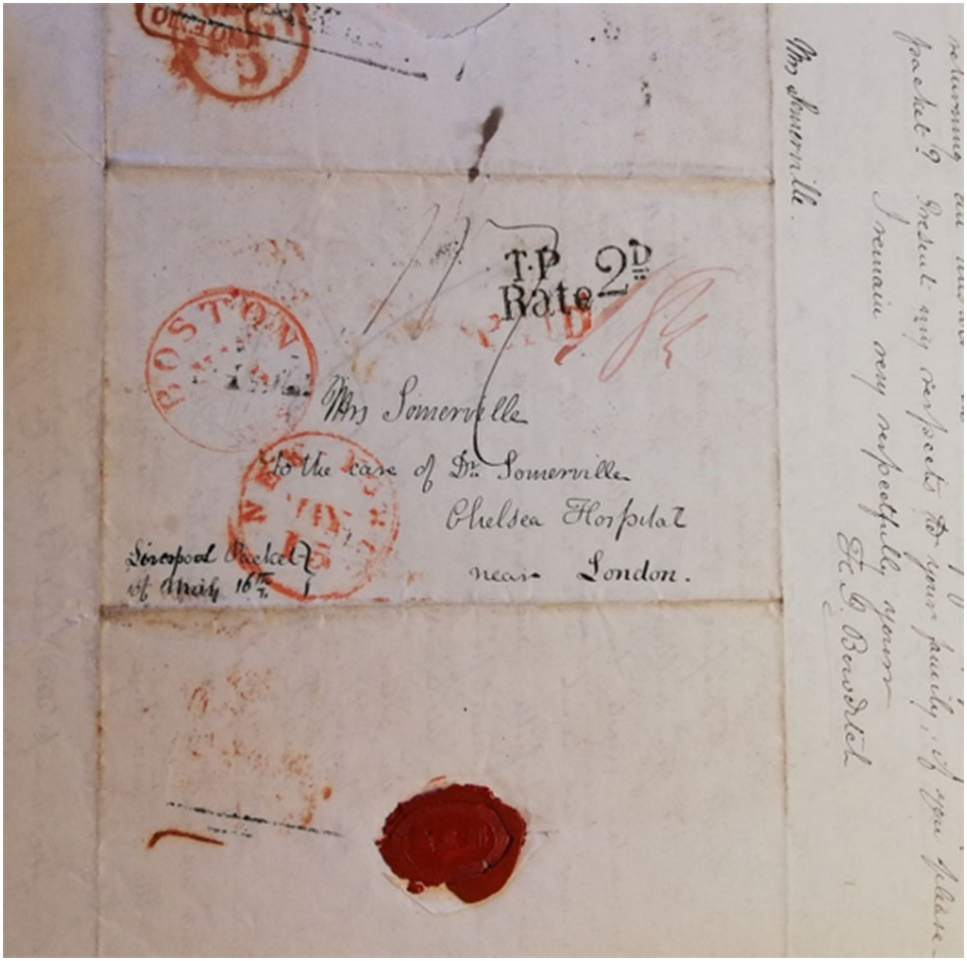
Letter of 1838 from Henry Bowditch addressed to “Mrs Somerville, to the care of Dr Somerville, Chelsea Hospital, near London.”

Although Somerville was a prolific letter writer and maintained a vast network of personal correspondents throughout Western Europe for much of her life, a significant proportion of her correspondence was mediated through her husband, not least because William’s increased visibility as a professional man, specifically surgeon general at the Royal Hospital, Chelsea, meant that he was more easily contactable than Somerville; if their personal address was unknown, letters could instead be addressed to that institution, to be forwarded.

On returning to the United States of America after being hosted in Chelsea by the Somervilles, Henry Ingersoll Bowditch (1808–1892, American physician and abolitionist) wrote directly to Mary Somerville but addressed the letter “Mrs Somerville, to the care of Dr Somerville, Surgeon of the Royal Chelsea Hospital.”^40^ In his letter, Bowditch updated Somerville on the progress of his father Nathaniel Bowditch’s own annotated translation of Laplace’s *Mécanique céleste* and, following on from a conversation with William at Chelsea, where Bowditch heard of her desire for a sample of “Green Feldspar,” sent a selection of minerals that he thought might be of interest to her. Bowditch sent a further three letters to Somerville via the Royal Chelsea Hospital; the last letter was again addressed “care of Dr Somerville” and was sent after a period of silence lasting three years (see Figure 5).^41^

**Figure 6:**
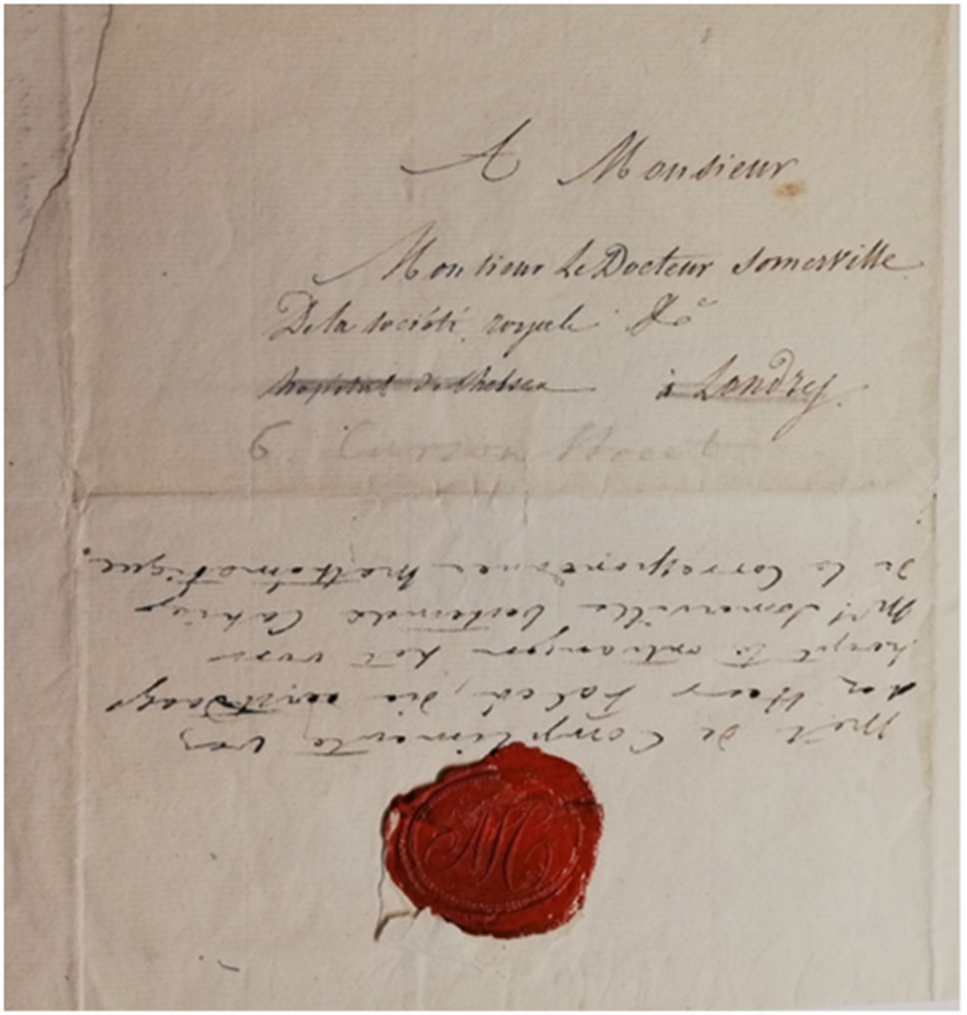
Letter from Adolphe Quetelet addressed to “Monsieur le Docteur Somerville de la société royale &c., hospital de Chelsea à Londres.”

Similarly, Adolphe Quetelet (1796–1874), a Belgian astronomer and mathematician whom the Somervilles had met while visiting Brussels in 1824, addressed his letter of 26 September 1827 to Dr. William Somerville at the Chelsea Hospital (see Figure 6); it was subsequently redirected to their rented accommodation in central London, 6 Curzon Street (written in pencil) [34, 51].^42^ Thus, in a community where families often had multiple houses or would change location for the social season, it was beneficial to have a permanent professional address to which letters could be sent.

In addition to being a reliable point of contact, William acted as a node through which books and papers could be passed to Somerville. Quetelet accompanied his aforementioned letter with the second volume of *Correspondance mathématique et physique* to be presented to Somerville as “a small token of respect for the talents and amiable qualities for which she is distinguished.”^43^ This volume was edited by Quetelet and contained a French translation of Somerville’s 1826 paper on magnetism, written by himself [39]. In another instance, the mathematician Augustus De Morgan sent William the volumes of Jean Sylvain Bailly’s *Histoire de l’astronomie moderne* [5], asking him to present them to “Mrs Somerville” and assure her that she can keep them as long as she would like.^44^

Two years before her election to honorary membership in the Royal Astronomical Society, at the Annual General Meeting of 1833, the council ordered the Greenwich Observations to be made available to Somerville to assist in her work. The Greenwich Observations, or *The Astronomical Observations Made at the Royal Observatory, Greenwich*, was a compendium of observations published annually under the remit of the astronomer royal. Both the Royal Society and the RAS were granted the privilege of distributing a number of copies as they saw fit; a list of recipients was printed in the *Memoirs* of the RAS, which included observatories and scientific institutions across Europe, India, and the USA, as well as around 50 individuals. Somerville’s name was included in Volume 5, in 1833, up until Volume 27, published in 1859, after which the lists stopped appearing [17].^45^ In the letter from Francis Baily of February 1833 (mentioned above), Baily informed William that all volumes of the Greenwich Observations printed so far were ready to be delivered to Somerville. Baily suggested that they be left for William at the Athenaeum Club, where he could collect them at his convenience and ensure their safe delivery to Somerville.^46^

## William Somerville as Literary Agent

During the 1830s, Somerville began using her acquired knowledge to supplement her income through the publication of books. Her husband thus began to take on a new role, as an informal literary agent. That is to say, William took charge of the correspondence with her publishers dealing with finances and accounts, and other business-oriented tasks necessary to publish a book [34, 117], [32, 69]. The professional role of the “literary agent” was not formalized until the late nineteenth century, but the gentlemen’s clubs in London had long been a space for those with literary aspirations to make “strategic friendships” or to further their business interests [21, 131,133]. Thus, as an active member of the Athenaeum Club and Royal Society, William was well placed to assist Somerville in becoming a published author.

Although Henry Brougham had been socially acquainted with Mary Somerville since the turn of the century, it was to William he wrote when seeking an author for a translation of Laplace’s *Mécanique céleste*. In his letter dated 27 March 1827, reproduced in [47, 161–162], Brougham informed William that he wished for an account of Laplace, in English, that explained its “vast merit, the wonderful truths unfolded or methodized—and the calculus by which all this is accomplished.” When Brougham subsequently decided that the account that Somerville had produced was too long and technical to be printed as part of his Library of Useful Knowledge as initially planned, it was William who then arranged for the work to be printed by John Murray (1778–1843, a fellow Scot and publisher of Sir Humphry Davy’s *Consolations in Travel* in 1830) [34, 75]. In addition, he sent some of the introductory sheets to Charles Babbage and solicited potential titles under which the work might be printed. Although Babbage was impressed by the pages he read, he was unable to offer any title suggestions in his reply to William.^47^

After much deliberation, the title *Mechanism of the Heavens* was decided upon, and the book appeared in print in November 1831. Around 70 copies were presented by Somerville to her friends and contemporaries [34, 118], many of whom replied with letters to *William* exclaiming their thanks and delight; Francis Baily thought the work invaluable for the “improvement” of the public and wished that he could soon pay his respects to Somerville in person.^48^ Editor of the *Edinburgh Review* Macvey Napier wrote to William to discuss arrangements for a review to appear in the March edition of said journal,^49^ and after hearing from John Herschel about Somerville’s “great work on the *Mécanique Céleste*,” Quetelet wrote to William to notify him that an announcement of the book would appear in *Correspondances Mathématiques et Physiques*.^50^

In his letter of thanks, again addressed to William, Henry Kater remarks that “Mrs Somerville has now publickly taken her station in science ... [which] is a very lofty one & such as no woman ever before reached.”^51^ Although the public and private spheres have often been identified as distinct and separate in nineteenth-century Britain, with women becoming more and more confined to the domestic private sphere during this time, Kater’s letter clearly highlights how the nature of Somerville’s presence in these spheres, like that of so many other middle- and upper-class women at the time, was anything but straightforward.^52^ Unable to fully engage in public scientific discourse through memberships in learned societies or appointments at universities or observatories, Somerville’s mathematical and scientific pursuits nonetheless relied upon and unfolded in both spheres.

Almost immediately after the publication of *Mechanism of the Heavens*, Somerville began preparing her next book. Although it contained no mathematical formulas, *Connexion of the Physical Sciences* [44] continued Somerville’s work in publicizing and advocating for the adoption of the mathematics contained within Laplace’s work. Indeed, many of the passages on physical astronomy are taken from her first book but are repurposed to demonstrate the fecundity of the mathematics without going into technical details. For example, when discussing the figure of the Earth, Somerville describes how “the moon’s eclipses show the earth to be round, and her inequalities not only determine the form, but the internal structure of our planet; results of analysis which could not have been anticipated” [44, 42]. In her conclusion, it is mathematical analysis that provides the “connexion” between the physical sciences, and will “ultimately embrace almost every subject in nature in its formulae” [44, 413].

William continued to assist Somerville in the preparation of this second book, consulting with Francis Baily over the formatting and typesetting of measurements, and sending sheets to William Whewell (1794–1866, former member of the Analytical Society and later master of Trinity College, Cambridge) to be proofread before publication [34, 130]. During her time in Paris between 1832 and 1833, Somerville had discussed her upcoming work with the new professor of natural history at the University of Edinburgh, James David Forbes (1809–1868). Since *Connexion* was not to be published until after the academic year had begun, Forbes reached out to William to request a manuscript copy of the work so he could give an account of it in his lectures.^53^ Two months later, Forbes wrote again, thanking William for sending him the sheets of “Mrs Somerville’s delightful book,” noting two corrections but refusing the request of writing a review for the *Quarterly Review*, citing his prior commitments.^54^ Again, these letters to William came after Forbes had written directly to Somerville earlier that same year and had obviously met her in person when they both visited Paris. Thus for matters of business, as the publication of her books was seen to be, many of Somerville’s correspondents preferred to communicate through her husband, who, it seems, was only too happy to oblige.

## Final Remarks

Viewing the Somervilles as a collaborative couple adds a wholly new perspective to existing literature on nineteenth-century scientific married couples. While [27] goes some way to deconstructing the pervasive husband-creator/wife-assistant narrative, nevertheless in the given case studies of heterosexual couples, it was the man who was the more visible, productive, or respected member of the partnership, especially when scientific labor was the primary focus. Moreover, Somerville’s close engagement with the scientific institutions of the day, which were nominally closed to women, adds greater depth to histories that usually focus on the “firsts” to overcome barriers to their inclusion—first woman to publish a paper, first woman to be elected a member, and so on.^55^ Through the collaboration of her husband, Somerville was able to engage meaningfully with the scientific communities centered on these institutions, over a century before the Royal Society eventually began electing women as members or Cambridge University granted women degrees.

The ways in which Somerville actively benefited from her marriage to William were multifaceted. With William to act as her willing chaperone, Somerville was able to travel more freely within society and across Europe, enabling her to engage personally with philosophers and savants throughout Western Europe. He also mediated much of Somerville’s correspondence, including the receipts of books and papers, and acted as a stable point of contact through his professional affiliations. Finally, as Somerville’s career as an author grew, William gained a new role in their relationship by taking ownership of the business-oriented tasks that were necessary to carry a book from conception to publication.

Therefore, although Somerville was precluded from being elected to memberships of learned academies and societies during her lifetime due to her gender, through the active support of her husband, Dr. William Somerville, she was nonetheless able to engage productively and meaningfully in the scientific and mathematical communities of which they formed a significant part. In fact, it was his cooperation, rather than her elections to honorary society memberships from the 1830s onward, that really enabled Somerville to circumvent gendered barriers to her engagement.

## Archival Resources and Acknowledgments

The majority of letters and certificates referenced here are held in the Mary Somerville Papers at the Bodleian Library, Oxford, on behalf of Somerville College. The box, folder, and page identifications are given in the text as, e.g., MS, Dep. c. 371, MSK-1 41, Mary Francis Kater to Mary Somerville 12/04/1832. Quotations and images are reproduced with the kind permission of the Principal and Fellows of Somerville College, and Sir Edmund Ramsay-Fairfax-Lucy, Bart.

Thanks also to the Mistress and Fellows, Girton College, Cambridge, for permission to quote from their Somerville Collection, and to the Royal Society of London for permission to quote from the Herschel Papers.

## Footnotes

1 A *conversazione* was an annual social gathering hosted by the Soirées Committee of the Royal Society at which experiments and objects of interest were displayed by and for fellows.

2 Women were first elected fellows of these societies in 1945 (Kathleen Lonsdale FRS and Marjory Stephenson FRS) and 1916 (Mary Adela Blagg FRAS, Ella Church FRAS, Alice Grace Cook FRAS, Irene Elizabeth Toye Warner FRAS, and Fiammetta Wilson FRAS) respectively.

3 The first woman elected to the London Mathematical Society was Charlotte Scott, in 1881, and Sofya Kovalevkaya was the first woman elected to the Société Mathématique de France, in 1882.

4 Throughout this paper, “polite” will be used to refer to the social status of the circles in which Somerville moved; namely the landed gentry [49, 13].

5 Another demographic often excluded from scientific institutions was people of low social status. The engagement of “artisans” with natural-historical knowledge through corresponding with gentlemen is treated in [41].

6 In 1787, 1794, and 1796. The first two of these letter extracts were written to the secretaries of the Royal Society at the time (Charles Blagden (1748–1820) and Joseph Planta (1744–1827) respectively), while the third was written to the president of the Royal Society, Joseph Banks (1743–1820). It is presumably these recipients who read the letters to the Royal Society.

7 Somerville was born Mary Fairfax, but she will be referred to throughout this article as Somerville, since that is the name under which she published and was known. This was, in fact, Somerville’s third name; in 1804 she had married her second cousin Samuel Greig and assumed the name Mary Greig. Unfortunately, we have very little information regarding her social connections to the scientific community during their marriage. Greig is briefly mentioned in [47], where, in stark contrast to William Somerville, he is described as very uninterested in and unsupportive of Somerville’s scientific pursuits. Greig died in 1807, leaving Somerville with two small sons, only one of whom would reach adulthood (Woronzow Greig, 1805–1865, a barrister who was elected Fellow of the Royal Society in 1833).

8 Certificates of memberships are held in MR, Dep. c. 375, along with further election certificates from later in her life.

9 MS, Dep. c. 375, MSDIP-2, John George Children (FRSec) to William Somerville 19/02/1832.

10 MS, Dep. c. 375, Folder MSDIP-3, Augustus De Morgan to Mary Somerville 13/02/1835.

11 MS, Dep. c. 370, Folder MSD-3 123, Augustus De Morgan to Mary Somerville 08/09/1844.

12 The publications of Somerville’s male scientific contemporaries often identified the numerous society affiliations of the author, for example [3, 19, 37].

13 An account of his expedition in Southern Africa was subsequently published as an appendix to [6]; William’s own writing on the topic was published in [11].

14 Member and Fellow were used interchangeably by the RAS [8, 20]. Membership was divided into ordinary members and honorary members, the latter of which were usually resident abroad.

15 William’s father, the Reverend Thomas Somerville, had been a member of the RSE since 1793 [50, 869].

16 At the time, election certificates of candidates were displayed at ten ordinary meetings of the society, and they required the signatures of three or more members to be successful [10, 168].

17 Election certificates available online at royalsociety.org.

18 John Playfair to William Herschel, Royal Society Herschel Papers, HS/14/169, 16/06/1812.

19 Maria Edgeworth to Miss Ruxton 17/01/1822, reproduced in [47, 156]

20 Election certificates were viewed at royalsociety.org/collections/ on 6/5/2020.

21 Meyer gives no insight as to how, or whether, female servants hired as companions for one-way journeys made it back home.

22 Traveling with children was not unusual at the time. In 1817, the Somervilles took their four-year-old daughter on their tour of the continent but left their two sons in the care of relatives in Scotland and their two infant daughters with William’s sister [34, 17–19]; seven years later, when traveling to the Low Countries, Somerville’s 19-year-old son from her first marriage accompanied them, but their two young daughters were left in the care of a governess (their eldest daughter having recently died) [34, 44–45].

23 MS, Dep. c. 371, MSJ-1 20, Francis Jeffrey to Mary Somerville 13/08/1834. For more information on the BAAS, see [30] and [13].

24 Unfortunately, a treatment of the real and perceived dangers for women traveling or navigating society without a suitable chaperone in the nineteenth century is beyond the scope of this paper.

25 Somerville’s copy of [22] was bequeathed to Girton College, Cambridge, where it is held today, along with the other mathematical texts Somerville owned at the time of her death [47, 80, 347]. It bears the inscription “Mary Somerville, from Professor Wallace.” Girton College Library: Somerville Collection (073119).

26 See the forthcoming PhD thesis from this author. In [47], Somerville mistakenly claims that she was not introduced to the Herschels until she moved to London in 1816. Somerville also notes that Caroline Herschel was abroad at the time of the visit and makes no mention of having met her on a subsequent occasion [47, 106]. However, Caroline Herschel’s memoir suggests that the two women were aware of each other’s astronomical work, and in 1835, Somerville wrote to Herschel on the occasion of their being simultaneously elected the first women honorary members of the RAS and offered a copy of her second book, *On the Connexion of the Physical Sciences* [45] [20, 274].

27 MS, Dep. c. 355, MSAU-1. Somerville’s diary covering her time in Paris is reproduced in [35]. When Humphry Davy traveled to Paris four years earlier, notably during the reign of Napoleon, he visited many of the same institutions as Somerville, including the Jardin des Plantes and the Institut de France. It appears that women were welcome to attend meetings of the Académie des Inscriptions et Belles-Lettres at the Institut de France as spectators, for Somerville did so in the company of Gabrielle Biot. This was a somewhat less triumphant welcome than that of Davy, who was seated at the right hand of the president of the first class of the Institut and whose presence was officially announced to the meeting [33, 14].

28 “L’intérêt que vous [Somerville] daignez prendre à mes ouvrages me flatte d’autant plus, qu’ils ont bien peu de semblables lecteurs et de juges aussi éclairés.”

29 This copy of Laplace’s *Système du monde* [24] is also held in the collection at Girton College, Cambridge (see footnote 24).

30 Sunday 30 December 1838, Lord Esher’s typescripts, viewed online at www.queenvictoriasjournals.org on 22/05/2020. Esher mistakenly wrote La Grange rather than La Place; however, Lagrange died in 1813, four years before Somerville visited Paris.

31 There were six types of subscriptions available, including an annual subscription offered only to “Ladies” that allowed access to the lectures and mineralogical collections, but not the library and model room [25, 59].

32
[25, 291] and Royal Institution Managers Minutes, 7 March 1825, volume 7, p. 11. I am extremely grateful to Frank James for providing the latter information and reference during the archival closures caused by Covid-19.

33 MS, Dep. c. 369, MSB-1 265, Charles Babbage to William Somerville (undated).

34 MS, Dep. c. 369, MSB-1 230 & 238. Babbage’s papers are “An Essay Towards the Calculus of Functions,” *Philosophical Transactions*, Charles Babbage, June 1815; “An Essay Towards the Calculus of Functions Part 2,” *Philosophical Transactions*, Charles Babbage, March 1816; “Observations on the Analogy Which Subsists between the Calculus of Functions and other branches of analysis,” *Philosophical Transactions*, Charles Babbage, April 1817; “Observations on the Notation Employed in the Calculus of Functions,” *Transactions of the Cambridge Philosophical Society*, Charles Babbage, May 1820; “On the Influence of Signs in Mathematical Reasoning,” *Transactions of the Cambridge Philosophical Society*, Charles Babbage, Dec. 1821, held in MS, Dep. c. 369, MSB-16.

35 The Analytical Society was founded in 1812 by a small group of Cambridge University students with a common interest in combating a perceived decline in British mathematics [14].

36 MS, Dep. c. 369, MSB-4 290, Francis Baily to William Somerville 03/02/1833.

37 It is unclear from the letter which council meeting this is; both William Somerville and William Broderip were involved in the Geological Society and the Linnean Society. The “council” of a learned society was the meeting of the fellows responsible for the running of the society, usually led by the president, treasurer and secretary.

38 MS, Dep. c. 369, MSB-12 367, William Broderip to William Somerville 15/03/1832.

39 As demonstrated by the multiple society memberships of most actors mentioned in this paper.

40 MS, Dep. c. 369, MSB-12 374, Henry Ingersoll Bowditch to Mary Somerville 24/2/1834.

41 Bowditch wished for Somerville to write a review of his father’s Laplace translation to be inserted in Nathaniel Bowditch’s biography.

42 MS, Dep. c. 372, MSQ-1 1, Adolphe Quetelet to William Somerville 26/09/1827.

43 As “un bien foible témoignage de respect pour les talens et les qualités aimables qui la distinguent,” MS, Dep. c. 372, MSQ-1 1, Adolphe Quetelet to William Somerville 26/09/1827.

44 MS, Dep. c. 370, MSD-3 126, Augustus De Morgan to William Somerville (undated).

45 In 1833, Somerville was the only woman listed, but the names of many of her correspondents also feature as recipients, including Nathaniel Bowditch, John Herschel, Henry Kater, and Adolphe Quetelet.

46 MS, Dep. c. 369, MSB-4 290, Francis Baily to William Somerville 03/02/1833. William was involved with the Athenaeum Club, a gentlemen’s club for those with scientific interests, from its foundation in 1824; the Athenaeum did not admit women as members until 2002.

47 MS, Dep. c. 269, MSB-1 229, Charles Babbage to William Somerville 02/09/1830.

48 MS, Dep. c. 369, MSB-4 289, Francis Baily to William Somerville 17/12/1831.

49 MS, Dep. c. 371, MSN-1 220, Macvey Napier to William Somerville 05/12/1831.

50 MS, Dep. c. 372, MSQ-1 3, Adolphe Quetelet to William Somerville 14/03/1832. The brief announcement was subsequently made in [40].

51 MS, Dep. c. 371, MSK-1 38, 23/04/1832, Henry Kater to William Somerville.

52 For more on women whose lives bridged the constructed separation between the public and private spheres in Georgian Britain, see [49].

53 MS, Dep. c. 370, MSF-2 231, James Forbes to William Somerville 20/08/1833.

54 MS, Dep. c. 370, MSF-2 233, James Forbes to William Somerville 21/10/1833.

55 See, for example, [7, 28].
